# A maximum likelihood algorithm for reconstructing 3D structures of human chromosomes from chromosomal contact data

**DOI:** 10.1186/s12864-018-4546-8

**Published:** 2018-02-23

**Authors:** Oluwatosin Oluwadare, Yuxiang Zhang, Jianlin Cheng

**Affiliations:** 1 Electrical Engineering & Computer Science Department, University of Missouri, Columbia, MO 65211 USA; 2 Informatics Institute, University of Missouri, Columbia, MO 65211 USA

**Keywords:** Hi-C, 3D chromosome structure, Gradient ascent, Chromosome conformation capture, 3D genome

## Abstract

**Background:**

The development of chromosomal conformation capture techniques, particularly, the Hi-C technique, has made the analysis and study of the spatial conformation of a genome an important topic in bioinformatics and computational biology. Aided by high-throughput next generation sequencing techniques, the Hi-C technique can generate genome-wide, large-scale intra- and inter-chromosomal interaction data capable of describing in details the spatial interactions within a genome. These data can be used to reconstruct 3D structures of chromosomes that can be used to study DNA replication, gene regulation, genome interaction, genome folding, and genome function.

**Results:**

Here, we introduce a maximum likelihood algorithm called 3DMax to construct the 3D structure of a chromosome from Hi-C data. 3DMax employs a maximum likelihood approach to infer the 3D structures of a chromosome, while automatically re-estimating the conversion factor (α) for converting Interaction Frequency (IF) to distance. Our results show that the models generated by 3DMax from a simulated Hi-C dataset match the true models better than most of the existing methods. 3DMax is more robust to structural variability and noise. Compared on a real Hi-C dataset, 3DMax constructs chromosomal models that fit the data better than most methods, and it is faster than all other methods. The models reconstructed by 3DMax were consistent with fluorescent in situ hybridization (FISH) experiments and existing knowledge about the organization of human chromosomes, such as chromosome compartmentalization.

**Conclusions:**

3DMax is an effective approach to reconstructing 3D chromosomal models. The results, and the models generated for the simulated and real Hi-C datasets are available here: http://sysbio.rnet.missouri.edu/bdm_download/3DMax/. The source code is available here: https://github.com/BDM-Lab/3DMax. A short video demonstrating how to use 3DMax can be found here: https://youtu.be/ehQUFWoHwfo.

## Background

A set of all chromosomes within the nucleus of a eukaryotic cell constitutes its genome. Studies of the organization of chromosomes and genomes reveal that they are structurally organized within a cell [1–3]. Studies find that this organization influences many biological mechanisms such as DNA replication, DNA repair, DNA translocation, gene regulation, transcription efficiency, genome interpretation, epigenetic modification, and genome stability maintenance [1–4]. The fluorescent in situ hybridization (FISH) [5] was often used in the investigation of the three-dimensional (3D) organization of a genome, but it cannot produce the layout of the genome structure at a large scale. The chromosome conformation capturing techniques such as 3C [6], 4C [7], 5C [8, 9], and Hi-C [10, 11] were developed to analyze the spatial organization of chromatin in a cell at a larger scale. The Hi-C technique can use next generation DNA sequencing to determine genome-wide spatial chromosomal interactions.

Much progress has been made in recent years on the study of chromosome and genome 3D structure modeling. Several methods have been proposed to construct the structure of an individual chromosome or an entire genome from chromosome conformation capturing data [12–24]. Some of these methods perform chromosome/genome 3D structure modeling in a two-step process, which involves converting interaction frequencies (IF) between fragment pairs in Hi-C data to distances between them, and then inferring the 3D structures that best satisfies the distances. Methods that implement this two-step process are known as distance restraint-based methods. Several of such methods have been proposed, each of which varies in restraint representation and optimization methods adopted [14–24].

In [12], Duan et al. considered the genome 3D structure prediction problem as a constrained non-convex optimization problem, and hence used an optimization solver (open-source software) IPOPT [25] to solve it. Bau et al. [13] also treated the 3D modeling problem as an optimization problem, and used the Integrated Modeling Platform (IMP) [26] to construct 3D structure models. The MCMC5C [14] method designed a probabilistic model for the interaction frequency (contact) data, and thereafter used a Markov Chain Monte Carlo (MCMC) approach to generate a representative structure from the data. ChromSDE [16] formulated the 3D structure modeling problem as a non-convex non-linear optimization problem, but then relaxed it as a semi-definite programming (SDP) problem. Bayesian 3D constructor (BACH) [17] is another method that employs MCMC to infer the 3D structure by maximizing the likelihood of the observed Hi-C data following a Poisson regression approach. MOGEN [19, 22] is a contact based method that is different from the rest, because it does not require the conversion of interaction frequencies to distances before structure construction. ShRec3D [24] is a two-step algorithm that uses the shortest path algorithm to realize chromosome structure construction. LorDG [27] uses a Lorentzian objective function to construct the 3D model of a chromosome or genome. Despite the significant progress made over the years, some of the distance-based chromosome structure modeling methods have several limitations: they may simply assume that the parameters used for converting interaction frequencies to distances are independent of input data and therefore are fixed for different datasets [8, 19], they may converge slowly (common for Markov chain Monte Carlo (MCMC) approach [28, 29]), and they sometimes require to adjust quite a few parameters [19, 22], making it difficult to use.

In this paper, we introduce a new method called 3DMax that uses a maximum likelihood approach to infer the 3D structures of a chromosome from Hi-C data. In the 3DMax algorithm, the conversion factor (α) parameter to convert IF to its distance equivalent is determined automatically from the data. We show that 3DMax is relatively faster than most of the existing methods, and it only depends on optimizing the structural coordinate of predicted models through the least square residuals. 3DMax is capable of translating contact data of a chromosome, or genome into an ensemble of probable 3D conformations to approximate the dynamic 3D genome structures of a population of cells of the same type. Our experiment also demonstrates how parameters such as the learning rate and the convergence constant (epsilon) can impact the performance of a constructed model. We also demonstrate the effect of using different normalization method on the different chromosome 3D structure prediction algorithms. We benchmarked 3DMax with several popular methods [13–16, 19], and the result showed that our method performed robustly in the presence of noise and structural variability. We applied our method to a synthetic chromosomal interaction dataset, and two experimentally generated Hi-C datasets: a karyotypically normal human lymphoblastic cell line (GM06990) and a malignant B-cell. We used the data from FISH experiments available for the cell lines as independent validations of the reconstructed 3D chromatin structures. We performed a comparative analysis of the performance of 3DMax and several existing 3D reconstruction methods on the Hi-C datasets normalized by three commonly used methods [30–32]. These experiments show that 3DMax is an effective method for reconstructing 3D chromosomal structures from Hi-C data.

## Methods

Generally, before Hi-C data [10, 11] are used for model construction, they are converted to a matrix form known as a contact matrix or a contact map.

### Chromosome contact map

A chromosome contact map is a N * N matrix, extracted from a Hi-C data, showing the number of interactions between chromosomal regions. The size of the matrix (N) is the number of equal-size regions of a chromosome. The length of equal-size regions (e.g. 1 Mb base pair) is called resolution. Each entry in the matrix contains a count of read pairs that connect two corresponding chromosome regions in a Hi-C experiment. Therefore, the chromosome contact matrix represents all the observed interactions between the regions (or bins) in a chromosome. The 3DMax algorithm takes as input a contact map to build the 3D structure of a chromosome.

### Structure initialization

To structurally represent a chromosome, each of its regions (or bins) is represented by three coordinates (*x, y, z*) in 3D space. In 3DMax, the structure construction starts with a random initialization of the coordinates of all the regions such that they are in the range [− 0.5, 0.5] as in [19].

### Maximum likelihood objective function of a chromosome structure

We used a log likelihood function as an objective function to compute chromosome structures from a contact map.

Let S stand for a 3D chromosome structure, and D represent the contact matrix data derived from a Hi-C dataset. The likelihood of S, P(D|S), can be expressed as the product of the probabilities of individual data points (interaction frequencies or distances) in D conditioned on the structure S, if the data points are conditionally independent of each other given a S. In 3DMax structure modeling, the input contact matrix is converted to spatial distances based on the assumption that the IF and the distance have an inverse relationship [14–18]. The conversion method is explained in the Subsection “*conversion of interaction frequency to spatial distance”* later. By assuming that data points *D*_*i*_ in D are conditionally independent given a structure S, we defined the likelihood (L(S)) in Eq. (1) as:

1 $$ \mathrm{L}\left(\mathrm{S}\right)=\mathrm{P}\left(\mathrm{D}|\mathrm{S}\right)={\prod}_{i=1}^nP\left({D}_i|S\right) $$

where n represents the total number of data points to be considered, and *D*_i_ represents the i^th^ data point (i.e., the distance between a pair of chromosomal regions derived from the contact matrix). Assumed that each data point i obeys the normal distribution, the probability of data point *D*_*i*_ can be described as:

2 $$ \mathrm{P}\left({\mathrm{D}}_{\mathrm{i}}|\mathrm{S}\right)\sim \frac{1}{\sigma \sqrt{2\uppi}}.\exp \left(-\frac{1}{2{\sigma}^2}{\left({D_i}^s-{D}_i\right)}^2\right) $$

where *D*_*i*_^*s*^ which is the actual Euclidean distance of the pair of regions corresponding to *D*_*i*_, computed from (*x,y,z*) coordinates of the two regions in 3D structure S as in [33]. *σ*^2^ is the variance of the distance. By combining Eqs. (1) and (2), we obtain the likelihood estimate of a structure S:

3 $$ L\ (S)={\left(\frac{1}{\sigma \sqrt{2\uppi}}\right)}^n.\exp \left(-\frac{1}{2{\sigma}^2}{\sum}_{i=1}^n{\left({D_i}^s-{D}_i\right)}^2\right) $$

By taking the logarithm of both sides of the Eq. (3), we obtain the log likelihood objective function in Eq. (4) for 3DMax chromosome structure reconstruction. Our goal is to find a structure S* that maximizes the likelihood function: L(S|D).

4 $$ L\ (S)=-\frac{\sum_{i=1}^n{\left({D_i}^s-{D}_i\right)}^2}{2{\sigma}^2}-n. log\sigma $$

With the assumption that the data is normally distributed according to Eq. (2), *σ* is calculated as in Eq. (5):

5 $$ \sigma =\sqrt{\frac{\sum_{i=1}^n{\left({Di}^s- Di\right)}^2}{n}} $$

We eliminated the dependence of the objective function on *σ* parameter by plugging Eq. (5) into the log likelihood objective function in Eq. (4). Hence, the resulting objective function L(S) can be represented as in Eq. (6). The objective function in Eq. (6) depends only on the (*x,y,z*) coordinates of regions in the structure.

6 $$ L\ (S)=-\frac{n}{2}-\mathrm{nlog}\sqrt{\frac{\sum_{i=1}^n{\left({Di}^s- Di\right)}^2}{n}} $$

### Gradient ascent optimization algorithm

We used the gradient ascent method to optimize the objective function iteratively until the 3DMax algorithm converges. 3DMax algorithm is considered converged, if the difference between the newly calculated log likelihood L(S) function value obtained with updated (x, y, z) coordinates and old L(S) function value of the previous step is less than a small constant value (epsilon). The determination of the epsilon value is described in the Results section.

Gradient ascent is an iterative optimization algorithm that moves in the direction of the function gradient. Using Eq. (6) as the base equation, we calculated the partial derivative of the log likelihood function with respect to a region’s x, y, and z coordinates in a 3D structure.

Once the partial derivative for each coordinate was obtained, we used the gradient ascent optimization method to adjust each coordinate to get a new structure S* that increases the likelihood. Equation (7) shows how the update was done, where λ is the learning rate, and S is the (x, y, z) coordinate vector in 3D space. If the learning rate is too small, it can result in a slow convergence to an optimal solution. But, if a larger learning rate is defined, the algorithm might oscillate around an optimal solution. There is no standard approach to choose λ value, but it is common to set a larger learning rate at the beginning of the optimization, and reduce it as the optimization progresses. The result of using the different types of learning rate is described in the Subsection “*choice of the learning rate*” in the Result section.

7 $$ {S}^{\left(t+1\right)}={S}^{(t)}+{\uplambda}^{(t)}\nabla L\left({S}^{(t)}\right) $$

where *t* is an iteration index, *S*^(*t*)^ is the structure coordinate at an iteration index t, λ^(*t*)^ is a learning rate at t that may vary as the iteration proceeds, and *∇L*(*S*^(*t*)^) is the partial derivative of the log likelihood with respect to the coordinates in the structure.

In this work, we also implemented a variant of the 3DMax algorithm above, called 3DMax1, which performs an extra pre-processing and filtering of the input contact matrix when the input is noisy (e.g. having low IFs). Moreover, 3DMax1 uses a stochastic gradient ascent algorithm with per-parameter learning rate, which is called the adaptive Gradient algorithm (AdaGrad). The AdaGrad [34] is a gradient-based optimization that can adapt the learning rate to each parameter, it performs larger updates for infrequent or sparse parameters and smaller updates for frequent or less sparse parameters. And it often improves convergence performance over standard stochastic gradient ascent when dealing with sparse parameters [35]. Different from 3DMax that updates the values of all the structure parameters in *S* at once with the same learning rate λ, AdaGrad in 3DMax1 uses a different learning rate for every parameter in *S* at every time *t* . Let Eq. (8) represent the gradient of the log likelihood for a parameter *S*_*i*_ at a time step t. Hence, the stochastic Gradient ascent in Eq. (7) can be written as in Eq. (9) for a parameter *S*_*i*_ in *S*.

8 $$ {g}_{t,i}=\nabla L\left({S_i}^{(t)}\right) $$

9 $$ {S_i}^{\left(t+1\right)}={S_i}^{(t)}+{\uplambda}^{(t)}.{g}_{t,i} $$

In the update rule for AdaGrad, it modifies the learning rate λ at each time step for every parameter *S*_*i*_ based on the previously computed gradient for the parameter *S*_*i*_. according to Eq. (10)

10 $$ {S_i}^{\left(t+1\right)}={S_i}^{(t)}+\kern0.5em \frac{\uplambda}{\sqrt{G_{t, ii}+\varepsilon }}.{g}_{t,i} $$

Here, *G*_*t*_ is a diagonal matrix where each diagonal element *i*, *i* is the sum of the squares of the gradients w.r.t. *S*_*i*_ up to time step t according to Eq. (11). While *ε* is a smoothing term that avoids division by zero (usually on the order of 1e − 6).

11 $$ {G}_t={\sum}_{i=1}^t\left({g}_i{g}_i\right) $$

In essence, *G*_*t*_ contains the sum of the squares of the past gradients for all the parameters in *S* along its diagonal. One of AdaGrad’s main benefits is that it eliminates the need to manually tune the learning rate at each iteration.

### Normalization of Hi-C data

Data normalization is necessary for Hi-C datasets, because there is a lot of noise in them. In this study, we used the iterative correction and eigenvector decomposition (ICE) technique [31] as the default technique to normalize the Hi-C data. The ICE technique was used to normalize the contact map derived from both the synthetic data and the experimental Hi-C data. The GM06990 Hi-C data was also normalized using the Yaffe and Tanay normalization technique [30]. The Yaffe and Tanay normalization technique normalizes the observed read counts by the expected read counts between the regions in a contact matrix. The other technique used to normalize the GM06990 Hi-C data is the Sequential Component Normalization (SCN) technique [32]. The results obtained by the three methods above are presented in the Results section.

### Conversion of interaction frequency to spatial distance

An important aspect of most distance restraint-based modeling approaches including 3DMax is to convert the interaction frequency (IF_ij_) between two regions (i, j) in a contact matrix to a hypothetical Euclidean distance. An inverse relationship is assumed to exist between them. The relationship is usually defined as 1/IF^α^, where IF is the interaction frequency, and α is called the conversion factor. According to [16], α cannot be too small because the spatial distance becomes independent of the interaction frequency as α approaches zero. And α also cannot be too large because in this situation a small change in interaction frequency could produce a significant difference in the spatial distances. Therefore, choosing a conversion factor that correctly represents the relationship between distance and interaction frequency (IF) is important. For 3DMax, we assume that the optimal α will be in the range [0.1, 2], which is consistent with the previous study [14, 16].

### Measurement of model similarity and accuracy

We used the Pearson correlation coefficient (PCC), the Spearman’s correlation coefficient (SCC), and the root mean square error (RMSE) to measure the similarities between chromosomal structures, and assess the accuracy of the constructed structures as in the previous studies [12–20]. When these assessment methods are applied on a distance representation of a model, or a distance representation of Hi-C data, they are sometimes called the distance Pearson Correlation Coefficient (dPCC), the distance Spearman Correlation Coefficient (dSCC), and the distance Root Mean Square error (dRMSE), respectively. For instance, if we have two pairwise distance dataset from two models, {d_i_, …, d_n_} containing n values, and another pairwise distance dataset {D_i_, …, D_n_} containing n values, the dPCC, the dSCC and the dRMSE can be computed using the formulas given below.

1. The distance Pearson correlation coefficient (dPCC) is defined as,
  $$ \mathrm{dPCC}=\frac{\sum_{i=1}^n\left({d}_i-\overline{d}\right)\left({D}_i-\overline{D}\right)}{\sqrt{\sum_{i=1}^n{\left({d}_i-\overline{d}\right)}^2{\sum}_{i=1}^n{\left({D}_i-\overline{D}\right)}^2}} $$

where:

- *d*_*i*_ and *D*_*i*_ are single distance samples indexed with i,
- *n* is the number of pairwise distance.
- $\overline{d}$ and $\overline{D}$represent sample means. $\overline{d}=\frac{1}{n}\sum \limits_{i=1}^n{d}_i$, $\overline{D}=\frac{1}{n}\sum \limits_{i=1}^n{D}_i$ .

(2) The distance Spearman’s correlation coefficient (dSCC) is defined as
  $$ \mathrm{dSCC}=\frac{\sum_{i=1}^n\left({X}_i-\overline{X}\right)\left({Y}_i-\overline{Y}\right)}{\sqrt{\sum_{i=1}^n{\left({X}_i-\overline{X}\right)}^2{\sum}_{i=1}^n{\left({Y}_i-\overline{Y}\right)}^2}} $$

dSCC is calculated by converting distance variable *d*_*i*_ and *D*_*i*_ into ranked variables *X*_*i*_ and *Y*_*i*_, and then, computing the dPCC between the ranked variables.

where:

- *X*_*i*_ and *Y*_*i*_ is the rank of two distance *d*_*i*_ and *D*_*i*_ respectively. Hence, X and Y is a vector of distance rank of the distance vector *d* and *D respectively*.
- $\overline{X}$ and $\overline{Y}$ represent sample means of rank. $\overline{X}=\frac{1}{n}\sum \limits_{i=1}^n{X}_i$, $\overline{Y}=\frac{1}{n}\sum \limits_{i=1}^n{Y}_i$ .

(3) The distance Root Mean Square Error (dRMSE) is defined as,
  $$ \mathrm{dRMSE}=\sqrt{\frac{1}{n}\sum {\left({d}_{ij}-{D}_{ij}\right)}^2} $$

- where *d*_*ij*_ and *D*_*ij*_ are the distance vector between regions i and j for the first model, and second model respectively.
- *n* is the number of pairwise distance.

The dSCC measures the similarity of the distance profiles of two 3D structures. The dSCC value varies between − 1.0 and 1.0; the higher the dSCC value is, the more similar the two structures are. It is worth noting that, to determine the dRMSE of two structures, the structures must be compared at the same scale. For instance, assuming two structures are represented with coordinates S**′** and S ∈ R ^n × 3^, where S**′** is the model constructed by 3DMax, S is the known model from a simulated data, and n is the number of regions representing a chromosome. To calculate the dRMSE value, we performed linear transformations that includes translation, orthogonal rotation, and rescaling of the points in the matrix R^3 x n^ of structure S**′** in order to best match them with the points in matrix R^3 x n^ of structure S. The Procrustes function library defined in MATLAB [36–39] is used to do the transformation of the dimensions. After the transformation, the dRMSE value between the scaled structure S″ and the original structure S is calculated.

### Datasets

The synthetic dataset from Trussart et al., 2015 [15] is a series of simulated Hi-C contact matrices where the genomic architectures are pre-defined and the noise level and structural variability (SV) are both simulated. The contact maps, the original models and their reconstructed models used in this study were downloaded from http://sgt.cnag.cat/3dg/datasets/.

The real Hi-C data used in this study is from a normal GM06990 cell line and a malignant B-cell line. The normal GM06990 dataset was downloaded from the Gene Expression Omnibus (GEO) repository under the accession number GSE18199. Its raw and normalized interaction frequency matrices at 1-MB resolution [10] were downloaded from [40]. We used the normalization pipeline described in [31, 32] to obtain normalized contact matrices. The raw contact matrices of the malignant B-cell 1-MB resolution were obtained from [41]. We used the pipeline [31] to normalize them. The fluorescence in-situ hybridization (FISH) data of the GM06990 cell line is from [10]. Its FISH distances and contact maps were obtained from [21].

## Results

We evaluated our method using a synthetic dataset (Trussart et al., 2015) [15] and two real Hi-C datasets of the two cell lines: a karyotypically normal human lymphoblastic cell line (GM06990) [10] and the malignant B-cell of an acute lymphoblastic leukemia patient [41].

### Parameter estimation

To use 3DMax, the conversion faction (α) needs to be defined. As the default, we set the α value to be in the range [0.1, 2] as explained in the Methods section. Another parameter we defined in 3DMax is the convergence constant called epsilon. To estimate the best epsilon value to use, we experimented on the GM06990_HindIII cell line dataset using six epsilon values, i.e., 1, 0.5, 0.1, 0.01, 0.0001, and 0.00001(Table 1). According to our experiment, although the different epsilons produced comparable dSCC average, the epsilon = 0.0001 has the highest average dSCC score. Hence, we set it as the default epsilon value for 3DMax. The number of ensemble structures (N) to generate per conversion factor is another parameter to be tuned. Table 2 shows the performance changes by setting different numbers of ensemble structures (NUM_STR). It is observed that a higher N value does not guarantee a significant increase in the accuracy. We set the default N to 5 in our implementation.

**Table 1:** The determination of the convergence constant (epsilon) values for the 3DMax algorithm

Chromosome	epsilon = 1	epsilon = 0.5	epsilon = 0.1	epsilon = 0.01	epsilon = 0.0001	epsilon = 0.00001
1	0.8087	0.8088	0.8087	0.8087	0.8088	0.8087
2	0.8149	0.8149	0.8149	0.8149	0.8149	0.8149
3	0.8306	0.8306	0.8306	0.8306	0.8306	0.8306
4	0.8716	0.8716	0.8714	0.8663	0.8735	0.8714
5	0.8645	0.8645	0.8645	0.8646	0.8654	0.8645
6	0.8477	0.8479	0.848	0.8478	0.848	0.848
7	0.8302	0.8302	0.83	0.8302	0.831	0.8301
8	0.8701	0.8701	0.8701	0.8702	0.8701	0.8701
9	0.853	0.853	0.8495	0.8521	0.8532	0.8508
10	0.8538	0.8542	0.8541	0.8538	0.8538	0.8538
11	0.8431	0.8431	0.8431	0.8431	0.8433	0.8432
12	0.8576	0.8576	0.8578	0.8577	0.8578	0.8578
13	0.8581	0.8553	0.8582	0.8582	0.8584	0.8582
14	0.8785	0.8796	0.8797	0.8797	0.8797	0.8797
15	0.8593	0.8563	0.8588	0.8595	0.8565	0.8592
16	0.8441	0.8459	0.8458	0.8459	0.8458	0.8458
17	0.8359	0.836	0.8362	0.8362	0.8362	0.8361
18	0.8521	0.8537	0.8536	0.8535	0.8535	0.8534
19	0.8629	0.8669	0.8663	0.8665	0.8665	0.8664
20	0.8853	0.884	0.8842	0.8865	0.8867	0.8867
21	0.9019	0.8995	0.9016	0.9016	0.9017	0.9018
22	0.8657	0.8658	0.8672	0.8658	0.8659	0.8659
Average dSCC	0.8541	0.8541	0.8543	0.8542	**0.8546**	0.8544

The dSCC value between the input distance matrix and the representative model for chromosome 1 – 22 of the GM06990 cell line using convergence constant (epsilon): 1, 0.5, 0.1, 0.01, 0.0001, and 0.00001 respectively. The average dSCC values across the chromosomes show that the results are highly comparable. The epsilon = 0.0001 has the highest average dSCC score, hence, we set it as the default epsilon value for 3DMax. The bold text represents the highest dSCC value

**Table 2:** The comparison of the performance when a constant learning rate and a decreasing learning rate are applied

	Constant Learning Rate		Decreasing Learning Rate	
Input Parameters	Running Time	Accuracy (Average dSCC)	Running Time	Accuracy (Average dSCC)
CHR = 1-22, NUM_STR = 1, ALPHA = constant	4 min	0.821	13 s	0.8493
CHR = 1-22, NUM_STR = 1, ALPHA = [0.1, 2]	1 h, 30 min	0.8456	3 min	0.8536
CHR = 1-22, NUM_STR = 5, ALPHA = [0.1, 2]	7 h	0.8546	20 min	0.8546
CHR = 1, NUM_STR = 1, ALPHA = constant	37 s	0.7556	2 s	0.8088
CHR = 1, NUM_STR = 5, ALPHA = [0.1, 2]	1 h	0.7841	3 min	0.8088
CHR = 21, NUM_STR = 1, ALPHA = constant	0.7 s	0.8969	0.2 s	0.8995
CHR = 21, NUM_STR = 5, ALPHA = [0.1, 2]	36 s	0.9018	2 s	0.9018
CHR = 21, NUM_STR = 30, ALPHA = [0.1, 2]	4 min	0.9018	12 s	0.9018
CHR = 21, NUM_STR = 50, ALPHA = [0.1, 2]	6 min	0.9021	18 s	0.9018
CHR = 21, NUM_STR = 100, ALPHA = [0.1, 2]	12 min	0.9020	37 s	0.9020
CHR = 21, NUM_STR = 200, ALPHA = [0.1, 2]	24 min	0.9022	83 s	0.9020
CHR = 21, NUM_STR = 500, ALPHA = [0.1, 2]	1 h	0.9022	3 min	0.9021

The comparison of the computing time and the average dSCC value obtained by using a constant or a decreasing learning rate for different input parameters for the chromosome 1 – 22 of the GM06990 cell line. We used the constant learning rate 0.0001, and we defined the initial_λ = 0.01 for the decreasing learning rate. CHR represents the chromosome number, and NUM_STR represents the number of ensemble structures generated per conversion factor(α), ALPHA represents the conversion factor. The decreasing learning rate achieved a better computing speed in all the cases

We executed all the other methods following the directions for parameter settings by their authors. All the parameters used to produce all the results are made available in the “parameters” directory of each method in the 3DMax website (http://sysbio.rnet.missouri.edu/bdm_download/3DMax/). For instance, to evaluate the MOGEN program, we used the parameters that produced the best result after trying multiple settings for the parameters required by the algorithm. The different parameters used to generate the MOGEN models, the input data, and the outputs for the three normalization methods for the GM06990 cell line are all available at the 3DMax website.

### Choice of the learning rate

As mentioned in the Methods section, the choice of the best learning rate can sometimes be a difficult task. However, it is common practice to use either a preferable constant learning rate, or a decreasing learning rate.

**Fig. 1:**
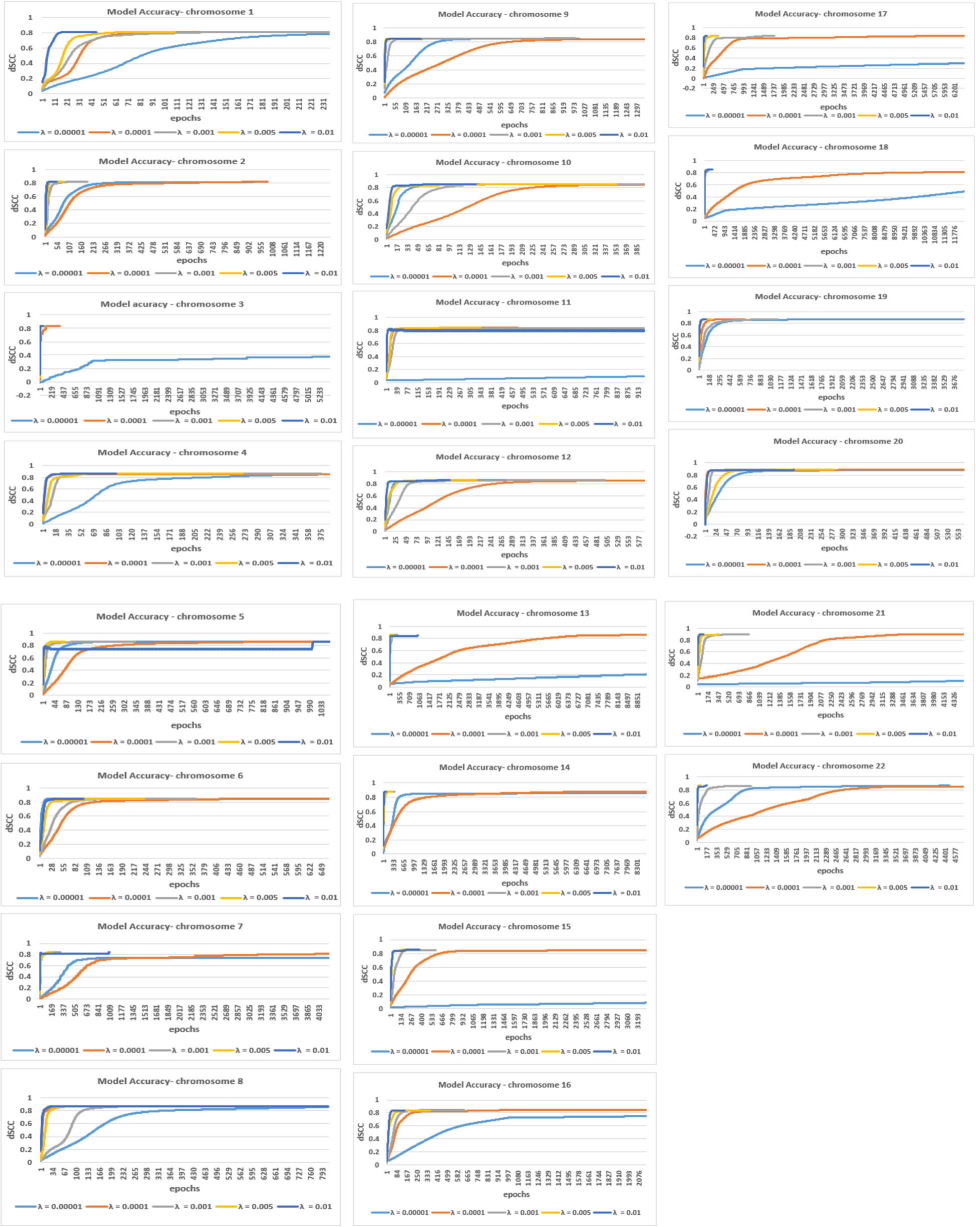
The comparison of the step by step model accuracy for different constant learning rate. The comparison of the dSCC model accuracy for five constant learning rates for GM06990_HindIII cell chromosome 1 to 22 dataset. We show the step by step dSCC till convergence for λ = 0.00001,0.0001, 0.001, 0.005 and 0.01 respectively for all the GM06990_cell chromosomes. The result shows that λ = 0.0001,0.001, and 0.005 had less fluctuations, and achieved a higher or similar dSCC value in cell chromosomes. Overall, the performance of 3DMax is comparable for each of the λ values. A higher dSCC value means the better accuracy

*The constant learning rate* uses a constant λ value through all the epoch steps for an algorithm. By experimenting with a range of learning rates in our work, Fig. 1 shows the model accuracy for different constant learning rates for GM06990_HindIII cell chromosome 1 to 22 datasets. The result shows the impact of using the different learning rates for structure modeling. We observed that λ = 0.0001, 0.001, and 0.005 shows a consistent better performance than the other λ values across all the chromosomes. As observed in the Figure, the larger learning rate (λ =0.01) had the advantage of faster convergence in some chromosomes, but suffered fluctuations or even decreased performance at some point (Chromosome 5,11, and 20). The smaller learning rates resulted in slow convergence and sometimes does not converge with a good model accuracy as in the case of λ = 0.00001 (Chromosome 3,11,13,15,16-18, and 21).

Conversely, for *the decreasing learning rate*, a typical way to implement it is to choose a starting learning rate, and drop the learning rate by half every 70 epochs (in our algorithm). This approach is termed the step based learning rate decay schedule. It takes the mathematical form below:

$$ \uplambda =\mathrm{initial}\_\uplambda \ast {0.5}^{\frac{1+ epoch}{70}} $$

**Fig. 2:**
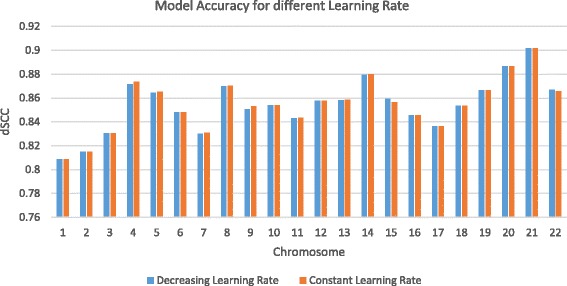
The comparison of the performance of 3DMax for constant and decreasing learning rates. Comparison of the result obtained by using the constant learning rate, and the decreasing learning rate shows that both methods achieved a comparable accuracy for all the chromosomes. A higher dSCC value means the better accuracy

In this work, we compared the result obtained by using the constant learning rate (λ =0.0001), and the decreasing learning rate methods in Fig. 2. Interestingly, the results show that both methods achieved a comparable accuracy for all the chromosomes. However, in terms of the computing speed, 3DMax is faster when the decreasing learning rate is used than when the constant learning rate is used. The running time and accuracy of the two methods of setting learning rates are reported in Table 2. In 3DMax, we made the decreasing learning rate approach the default because it converges faster.

### Assessment on simulated datasets

The synthetic dataset includes a series of Hi-C matrices simulated from the pre-defined chromosome structures with different noise levels and structural variability (SV) level. Each worm like chain chromosome structure has ~ 1 Mb base pairs and is represented by 202 regions of 5 Kb base pairs each. The simulated data can be classified into two categories based on the different architectures of the chromosome structures: Topological Associated Domains (TAD)-like architecture and Non-Topological Associated Domains (Non-TAD)-like architecture [42–45]. Each of these architectures has three structural density levels (40 bp/nm, 75 bp/nm and 150 bp/nm), resulting in six density-architecture combinations. The entire synthetic dataset contains 168 simulated Hi-C matrices in total, i.e., six different combinations of density and architectures times seven levels of structural variability (SV) (denoted as 0, 1, 2, 3, 4, 5, 6) times four noise levels (i.e. 50, 100, 150 and 200). There are 28 simulated Hi-C contact matrices for each of the six density-architecture combinations. According to [14], the most difficult architecture to reconstruct is the 150 bp/nm density with no TAD-like features because of its higher resolution and lack of regular TAD sub-structures.

**Fig. 3:**
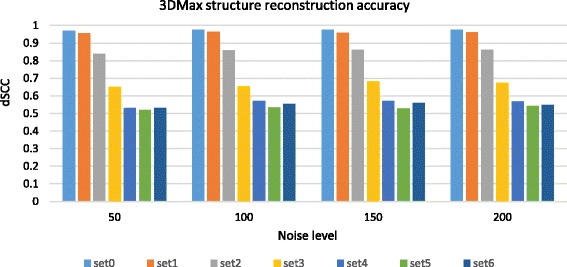
The dSCC accuracy of the structures generated by 3DMax for the synthetic data. The dSCC accuracy of the structures generated by 3DMax at different levels of noise and structural variability for conversion factor (α) = 0.3. The dataset has resolution 150 bp/nm and TAD like feature architecture. Y-axis denotes the distance Spearman correlation coefficient (dSCC) score in the range [− 1,1] and the X-axis denotes the noise level. Set 0-6 denotes seven different levels of structural variability in the increasing order. A higher dSCC value means the better accuracy

We evaluated 3DMax on the 28 contact matrices (7 levels of structural variability with four noise levels each) of the synthetic dataset with resolution 150 bp/nm for both TAD and non-TAD like feature architecture, respectively. The matrices were normalized with the ICE technique before they were used as input for 3DMax. To determine the best conversion factor (α) for model reconstruction, the dSCC value between the distance matrix generated from the input contact matrix and the Euclidean distance of the representative chromosomal model is computed. To determine the representative structure for an input matrix, we generated an ensemble of 50 structures and calculated the similarity between each structure in the ensemble with the input distance matrix. The structure with the highest dSCC value in the ensemble was chosen as the representative structure for the input contact matrix. We then computed the average dSCC value across the 28 contact matrices of the simulated data, with resolution 150 bp/nm and TAD like feature architecture, for the conversion factor (α) in the range [0.1, 2] (Table 3). The result shows that α value 0.3 has the highest average dSCC value. We computed the average dSCC value between the models reconstructed by 3DMax and the true structures (i.e., a set of 100 true structures for each structural variability level in the simulated dataset) for the α values in the range [0.1, 2] for the simulated data with resolution 150 bp/nm and TAD like feature architecture (Table 4). The result also shows that the structures generated at α = 0.3 have the higher similarity to the true structures from simulated dataset than other α values. To compute the accuracy of 3DMax, we compared each structure in the generated ensemble with the true structures (i.e., a set of 100 true structures for each structural variability level) by using the spearman correlation coefficient. We thereafter selected the reconstructed structure closest to a true structure from the ensemble. The spearman correlation coefficient of the selected structure and the true structure was averaged and used as the dSCC accuracy for the ensemble of generated 3DMax structures. The reconstruction accuracy (dSCC) for 3DMax at different levels of noise and structural variability (SV) for α = 0.3 shows that the accuracy of reconstructed models decreased as the structural variability level increased for each noise level (Fig. 3). The reconstruction accuracy of structures generated by 3DMax is relatively high for different noise levels when the structural variability (SV) is low, while the average accuracy of structures decreases noticeably as the level of SV increases.

**Table 3:** The average dSCC value between the distance matrix and the representative model for 28 contact matrices with different conversion factor (α) values

Conversion factor(α)	0.1	0.3	0.5	1.0	1.5	2.0
dSCC	0.759	**0.768**	0.758	0.695	0.638	0.559

The average dSCC value between the input distance matrix and the representative model for 28 contact matrices (7 levels of structural variability with four noise levels each) for the conversion factor (α): 0.1, 0.3, 0.5, 1.0, 1.5 and 2.0 respectively. The dataset has resolution 150 bp/nm and TAD like feature architecture. The bold text represents the highest dSCC value

**Table 4:** The average dSCC value for the dataset with resolution 150 bp/nm and TAD like feature architecture

Conversion factor(α)	0.1	0.3	0.5	1.0	1.5	2.0
dSCC	0.564	**0.720**	0.697	0.650	0.650	0.495

The average dSCC value between 3DMax model and the known structure for 28 contact matrices (7 levels of structural variability with four noise levels each) for the conversion factor (α): 0.1, 0.3, 0.5, 1.0, 1.5 and 2.0 respectively. The dataset has resolution 150 bp/nm and TAD like feature architecture. The bold text represents the highest dSCC value

Similarly, we evaluated 3DMax on 28 contact matrices of the synthetic dataset with resolution 150 bp/nm and non-TAD like feature architecture. Table 5 shows the performance of 3DMax for different α values.

**Table 5:** The average dSCC value for the dataset with resolution 150 bp/nm and non-TAD like feature architecture

Conversion factor(α)	0.1	0.3	0.5	1.0	1.5	2.0
dSCC	0.583	**0.658**	0.634	0.566	0.518	0.429

The average dSCC value between 3DMax model and the known structure for 28 contact matrices (7 levels of structural variability with four noise levels each) for the conversion factor (α): 0.1, 0.3, 0.5, 1.0, 1.5 and 2.0 respectively. The dataset has resolution 150 bp/nm and non-TAD like feature architecture. The bold text represents the highest dSCC value

### Comparison with existing methods on the simulated data

**Fig. 4:**
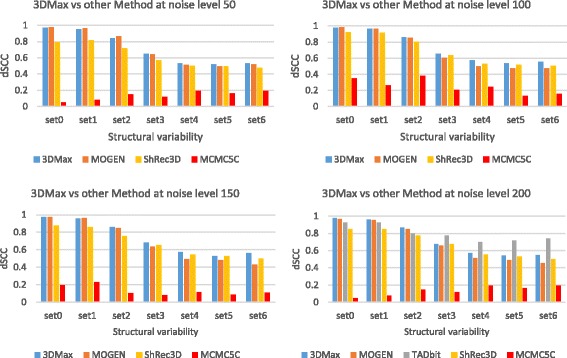
A comparison of the reconstruction accuracy of different methods on the synthetic dataset. The reconstruction accuracy for 3DMax, MOGEN, ShRec3D, and MCMC5C at different levels of noise and structural variability. The dataset has resolution 150 bp/nm and TAD like feature architecture. Top-Left: comparison at Noise Level 50, Top-Right: comparison at Noise Level 100, Bottom-Left: comparison at Noise Level 150, Bottom-Right: comparison at Noise Level 200. Y-axis denotes the distance Spearman correlation coefficient (dSCC) score in the range [− 1,1] and the X-axis denotes the structural variability level. Set 0-6 denotes seven different levels of structural variability in the increasing order. A higher dSCC value means the better accuracy

We compared 3DMax with three existing methods: MCMC5C [14], MOGEN [19], and ShRec3D [24]. We used each method to generate an ensemble of 50 structures for each input matrix. We compared each structure in the ensemble with the true structures (i.e., a set of 100 true structures for each structural variability level) using spearman correlation coefficient to select the reconstructed structure closest to a true structure from the ensemble. The spearman correlation coefficient of the selected structure and the true structures is averaged and used as the dSCC accuracy for the method. For clarity, the comparison is grouped based on the noise level of the simulated data from 50 to 200. For the different noise levels, 3DMax is comparable to the top method - MOGEN when structural variability (sets 0-1) is low. And as the variability increases (especially sets 3-6), it outperforms all the other methods (Fig. 4) most time. Table 6 shows a tabular representation of the dSCC values visualized in Fig. 4, to show the dSCC values generated by all the algorithms.

**Table 6:** A comparison of the reconstruction accuracy spread of the different methods on the synthetic dataset

SV	Noise Level 50			
	3DMax	MOGEN	ShRec3D	MCMC5C
set0	0.9708	0.9755	0.7928	0.0481
set1	0.9552	0.9648	0.8188	0.0779
set2	0.8405	0.8625	0.7175	0.1477
set3	0.6505	0.6406	0.5722	0.1201
set4	0.5302	0.5135	0.502	0.1916
set5	0.5211	0.4945	0.4938	0.1614
set6	0.5303	0.5211	0.4767	0.1938
SV	Noise Level 100			
	3DMax	MOGEN	ShRec3D	MCMC5C
set0	0.9753	0.9835	0.9239	0.3514
set1	0.963	0.968	0.9133	0.2642
set2	0.8578	0.8527	0.8072	0.3792
set3	0.6555	0.6039	0.6338	0.2068
set4	0.5703	0.4991	0.532	0.2456
set5	0.5342	0.4728	0.5183	0.1299
set6	0.5535	0.47	0.5026	0.1578
SV	Noise Level 150			
	3DMax	MOGEN	ShRec3D	MCMC5C
set0	0.976	0.9734	0.876	0.1933
set1	0.959	0.96	0.8613	0.2275
set2	0.8612	0.8485	0.7572	0.1016
set3	0.6821	0.6362	0.6546	0.0791
set4	0.5713	0.4915	0.5475	0.1146
set5	0.5285	0.4835	0.5268	0.0858
set6	0.5601	0.4318	0.5009	0.1106
SV	Noise Level 200			
	3DMax	MOGEN	ShRec3D	MCMC5C
set0	0.9771	0.9655	0.8499	0.0481
set1	0.9627	0.9533	0.8481	0.0779
set2	0.8634	0.8514	0.7743	0.1477
set3	0.6724	0.6606	0.6726	0.1201
set4	0.5679	0.5131	0.5559	0.1916
set5	0.5435	0.4886	0.5292	0.1614
set6	0.5487	0.4554	0.4992	0.1938

The reconstruction accuracy for 3DMax, MOGEN, ShRec3D, and MCMC5C at different levels of noise and structural variability. The dataset has resolution 150 bp/nm and TAD like feature architecture. Noise Level 50: comparison of dSCC value at Noise Level 50, Noise Level 100: comparison of dSCC value at Noise Level 100, Noise Level 150: comparison of dSCC value at Noise Level 150, Noise Level 200: comparison of dSCC value at Noise Level 200. The table values denote the distance Spearman correlation coefficient (dSCC) score in the range [−1,1] and the SV denotes the structural variability level. Set 0-6 denotes seven different levels of structural variability in the increasing order. A higher dSCC value means the better accuracy

### Assessment on real Hi-C data

**Fig. 5:**
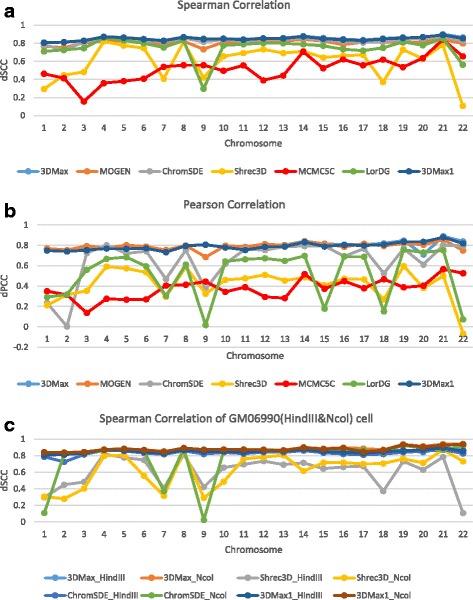
A comparison of the accuracy of different methods on real Hi-C datasets. **a** The Spearman Correlation Coefficient of 3DMax, 3DMax1, MOGEN, ChromSDE, ShRec3D, MCMC5C, and LorDG on the normalized contact maps of GM06990_HindIII cell. **b** The Pearson Correlation Coefficient of 3DMax, 3DMax1, MOGEN, ChromSDE, ShRec3D, MCMC5C, and LorDG on the normalized contact maps of GM06990_HindIII cell. **c** The Comparison of 3DMax, 3DMax1, ChromSDE and ShRec3D on the normalized contact maps of GM06990 HindIII and Ncol cell. Y-axis denotes either the distance Spearman correlation coefficient (dSCC) score in the range [− 1,1] or the distance Pearson correlation coefficient score (dPCC) in the range [− 1,1]. X-axis denotes the Chromosome number. A higher dSCC value means the better accuracy

We applied 3DMax to a 1 MB resolution Hi-C dataset of GM06990 cell line [10]. The Hi-C data for this cell line was generated with two different restriction enzymes: Ncol and HindIII. For comparison, we applied seven structure prediction methods 3DMax, 3DMax1 based on AdaGrad optimization algorithm, ShRec3D, ChromSDE, MCMC5C, MOGEN, and LorDG [27] to predict the 3D structure of chromosomes of this cell line. All the methods take as input an interaction frequency matrix normalized by using the normalization pipeline in [29]. We used the distance Spearman Correlation Coefficient (dSCC) and the distance Pearson Correlation Coefficient (dPCC) to assess the accuracy of these methods. The accuracy is determined by computing the dSCC value between the distance matrix of the normalized frequency input matrix and the Euclidean distance calculated from the predicted 3D structures. Figure 5(a) shows that 3DMax outperforms the other methods by at least 4% across 22 pairs of non-sex chromosomes of the cell line. 3DMax obtained an average spearman correlation coefficient of 0.85 across all the chromosomes while the second highest among the other methods has the coefficient of 0.82. Figure 5(b) shows the Pearson correlation coefficient on the GM06990_HindIII cell. 3DMax obtained the highest average Pearson correlation coefficient of 0.795, which is better than the other methods.

In Fig. 5(c) we compared the spearman correlation values of ShRec3D, ChromSDE, 3DMax, and 3DMax1 for the contact maps of GM06990 cell line with NcoI and HindIII restriction enzymes. 3DMax has the highest average dSCC value of 0.88 across the chromosomes of the cell line. Table 7 shows a tabular representation of the model accuracy comparison visualized in Fig. 5.

**Table 7:** A comparison of the accuracy spread of the different methods on real Hi-C datasets

	Spearman Correlation							
Chromosome	3DMax		3DMax1	MOGEN	ChromSDE	Shrec3D	MCMC5C	LorDG
1	0.8088		0.8062	0.7662	0.7845	0.2951	0.463	0.7101
2	0.8149		0.8126	0.7526	0.7245	0.4482	0.4143	0.7275
3	0.8306		0.828	0.8044	0.814	0.4827	0.1564	0.7459
4	0.8736		0.8715	0.8245	0.8636	0.8203	0.3595	0.8607
5	0.8653		0.8631	0.8266	0.8551	0.7762	0.3813	0.8317
6	0.848		0.845	0.8104	0.8303	0.7465	0.4078	0.8002
7	0.831		0.8278	0.7925	0.8144	0.4087	0.5402	0.7536
8	0.8701		0.8675	0.8236	0.857	0.8152	0.5584	0.8317
9	0.851		0.846	0.7339	0.8184	0.421	0.5584	0.2972
10	0.854		0.8505	0.8129	0.8392	0.6561	0.4967	0.7759
11	0.8433		0.8398	0.8003	0.823	0.6936	0.5559	0.7896
12	0.8558		0.8544	0.8259	0.8413	0.7332	0.3907	0.803
13	0.8584		0.8537	0.8242	0.8381	0.6917	0.4437	0.8007
14	0.8799		0.8754	0.8425	0.8605	0.7123	0.7065	0.7879
15	0.8592		0.8488	0.8255	0.8346	0.6432	0.5246	0.7725
16	0.8466		0.8397	0.7854	0.8188	0.6621	0.6208	0.7345
17	0.837		0.8298	0.8127	0.8083	0.6732	0.557	0.719
18	0.8537		0.8475	0.8139	0.8185	0.3717	0.6197	0.7492
19	0.8668		0.8579	0.8077	0.8397	0.73	0.5362	0.8152
20	0.8392		0.869	0.8146	0.8527	0.6291	0.6361	0.7779
21	0.9017		0.8925	0.8421	0.8704	0.7831	0.841	0.8532
22	0.866		0.8542	0.7977	0.8264	0.1065	0.6554	0.5639
	Pearson Correlation							
Chromosome	3DMax		3DMax1	MOGEN	ChromSDE	Shrec3D	MCMC5C	LorDG
1	0.7611		0.7491	0.7697	0.2352	0.2125	0.3497	0.2922
2	0.7511		0.7401	0.7544	0.0042	0.3154	0.314	0.3187
3	0.7603		0.7532	0.7938	0.7238	0.3539	0.1368	0.5597
4	0.7813		0.7691	0.7739	0.8016	0.5922	0.2758	0.6675
5	0.779		0.7661	0.8021	0.7215	0.5732	0.2673	0.6845
6	0.7834		0.7709	0.7883	0.7422	0.5361	0.2705	0.5945
7	0.7471		0.7334	0.7549	0.4693	0.2961	0.405	0.3044
8	0.7994		0.794	0.7988	0.7533	0.5895	0.4138	0.6126
9	0.8046		0.8063	0.6852	0.3711	0.3253	0.4446	0.017
10	0.7836		0.7793	0.7965	0.6214	0.4614	0.3436	0.6428
11	0.7628		0.7542	0.7852	0.7624	0.4761	0.39	0.6636
12	0.8098		0.7856	0.813	0.7533	0.5106	0.2941	0.6727
13	0.8037		0.7887	0.7989	0.7875	0.4544	0.2824	0.6483
14	0.8411		0.8316	0.8357	0.7928	0.4855	0.518	0.6965
15	0.8137		0.7892	0.8165	0.7948	0.4078	0.3745	0.179
16	0.8075		0.804	0.7845	0.6925	0.4726	0.4489	0.6899
17	0.8069		0.7981	0.8164	0.768	0.4662	0.3793	0.6879
18	0.82		0.8079	0.7931	0.5246	0.2697	0.468	0.1519
19	0.847		0.8356	0.8204	0.7674	0.5972	0.3881	0.7552
20	0.7096		0.8347	0.8049	0.6113	0.3825	0.4039	0.731
21	0.8892		0.8784	0.8561	0.802	0.5	0.5663	0.7509
22	0.8396		0.8181	0.7486	0.7958	−0.067	0.5262	0.0737
	Spearman Correlation Of GM06990 (HINDIII &NCOL) Cell							
Chromosome	3DMax_HindIII	3DMax_Ncol	3DMax1_HindIII	3DMax1_Ncol	Shrec3D_HindIII	Shrec3D_Ncol	ChromSDE_HindIII	ChromSDE_Ncol
1	0.8088	0.8432	0.8062	0.8412	0.2951	0.3043	0.7845	0.1085
2	0.8149	0.8387	0.8126	0.8367	0.4482	0.2797	0.7245	0.8228
3	0.8306	0.8447	0.828	0.8425	0.4827	0.403	0.814	0.8271
4	0.8736	0.874	0.8715	0.872	0.8203	0.796	0.8636	0.8624
5	0.8653	0.8836	0.8631	0.8816	0.7762	0.8077	0.8551	0.872
6	0.848	0.8701	0.845	0.8677	0.7465	0.556	0.8303	0.8539
7	0.831	0.8509	0.8278	0.8483	0.4087	0.3147	0.8144	0.3709
8	0.8701	0.8509	0.8675	0.8924	0.8152	0.8559	0.857	0.8832
9	0.851	0.8732	0.846	0.8721	0.421	0.2899	0.8184	0.0239
10	0.854	0.8753	0.8505	0.8723	0.6561	0.4865	0.8392	0.8603
11	0.8433	0.876	0.8398	0.8731	0.6936	0.76	0.823	0.8603
12	0.8558	0.873	0.8544	0.8698	0.7332	0.7819	0.8413	0.8531
13	0.8584	0.8665	0.8537	0.8621	0.6917	0.8064	0.8381	0.8457
14	0.8799	0.9	0.8754	0.8965	0.7123	0.6141	0.8605	0.887
15	0.8592	0.8842	0.8488	0.879	0.6432	0.715	0.8346	0.8707
16	0.8466	0.8975	0.8397	0.8921	0.6621	0.7156	0.8188	0.8856
17	0.837	0.8858	0.8298	0.8473	0.6732	0.6988	0.8083	0.866
18	0.8537	0.8701	0.8475	0.865	0.3717	0.7055	0.8185	0.8407
19	0.8668	0.936	0.8579	0.9324	0.73	0.7613	0.8397	0.925
20	0.8392	0.9133	0.869	0.9037	0.6291	0.7128	0.8527	0.8878
21	0.9017	0.9382	0.8925	0.9274	0.7831	0.873	0.8704	0.8688
22	0.866	0.9414	0.8542	0.9359	0.1065	0.7311	0.8264	0.922

Top: The Spearman Correlation Coefficient of 3DMax, 3DMax1, MOGEN, ChromSDE, ShRec3D, MCMC5C, and LorDG on the normalized contact maps of GM06990_HindIII cell, and the Pearson Correlation Coefficient of 3DMax, 3DMax1, MOGEN, ChromSDE, ShRec3D, MCMC5C, and LorDG on the normalized contact maps of GM06990_HindIII cell. Bottom: The Comparison of dSCC values of 3DMax, 3DMax1, ChromSDE and ShRec3D on the normalized contact maps of GM06990 HindIII and Ncol cell

The values denote the distance Spearman correlation coefficient (dSCC) score in the range [−1,1] or the distance Pearson correlation coefficient score (dPCC) in the range [−1,1]

On average, 3DMax’s accuracy is at least 3% higher than the other methods. In addition, since each Hi-C data obtained with a restriction enzyme is an independent observation of the GM06690 cell, we checked the robustness of our method by comparing the predicted structure from Ncol with one from the HindIII enzyme. We compared the predicted structure of chromosome 19 of HindIII data and NcoI replicate data. The dSCC and dRMSE value of the comparison were 0.9 and 0.0064 respectively, suggesting the two models are very similar.

### Consistency checking of models in ensembles

**Fig. 6:**
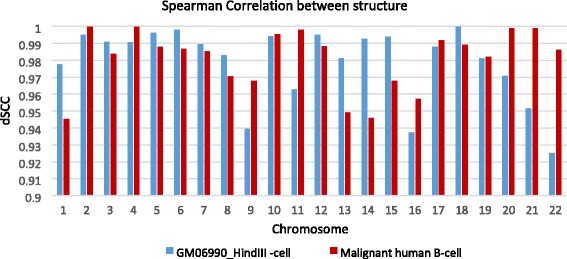
The similarity between structures generated by 3DMax. The average similarity for an ensemble of structures generated for the GM06990_HindIII cell and the malignant B-cell chromosomes using the optimal α value for each chromosome

To assess the consistency of the structures generated by 3DMax, we compared 50 structures generated at the optimal α value for each chromosome for the GM06990_HindIII cell and the malignant B-cell, respectively. We used the dSCC value to measure the similarity between these structures. Figure 6 shows the average dSCC for each chromosome for Hi-C data of the GM06990_HindIII cell and the malignant B-cell respectively. The average dSCC between the models is > 0.9 for all the chromosomes, indicating chromosomal models generated by 3DMax are quite similar to each other.

### Comparative analysis of the performance of 3DMax, 3DMax1, MOGEN, ChromSDE, ShRec3D, MCMC5C, and LorDG on Hi-C data normalized with three popular normalization methods

Due to biases in Hi-C experiments, Hi-C data is generally noisy. Some of these biases are associated with cutting frequencies of restriction enzymes, GC content and sequence uniqueness [11, 30–32]. In order reduce the effects of these biases, the Hi-C data contact matrix is normalized to reflect the strength of the underlying chromosomal interactions more accurately.

We performed a comparative study of the performance of different 3D modeling methods when each of the three commonly used normalization techniques: Yaffe and Tanay [30] normalization technique, ICE (iterative correction and eigenvector decomposition) technique [31], and Sequential Component Normalization (SCN) technique [32] is applied. Figure 5(a) shows the result obtained by using the Yaffe and Tanay normalization technique, where 3DMax outperformed the other methods. Table 8 shows the average dSCC value for different chromosomes for each of the normalization technique. 3DMax and 3DMax1 produces the best performance when the Yaffe and Tanay normalization technique is used, and the 3DMax1 produces the best performances when the ICE and SCN normalization method are used respectively. It is evident from the results that the normalization techniques have a significant impact on the performance of some 3D modeling methods.

**Table 8:** The average dSCC score of the chromosomal models of the GM06990 cell line reconstructed with three normalization techniques

	3DMax	3DMax1	MOGEN	ChromSDE	Shrec3D	MCMC5C	LorDG
Yaffe &Tanay	**0.85**	**0.85**	0.81	0.82	0.60	0.52	0.75
ICE	0.75	**0.85**	0.61	**0.83**	0.60	0.032	0.78
SCN	0.72	**0.85**	0.58	**0.83**	0.71	0.028	0.79

The average dSCC scores of chromosomal models of the GM06990 cell line reconstructed by 3DMax, 3DMax1, MOGEN, ChromSDE, ShRec3D, MCMC5C, and LorDG with the three normalization methods. The top 2 scores for each normalization technique are highlighted in bold text

## Discussion

### Comparison of the computing performance of the different methods

**Fig. 7:**
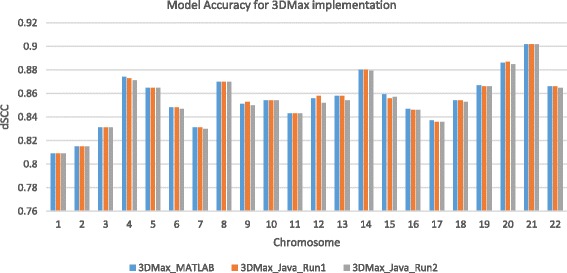
A comparison of the performance of 3DMax algorithm MATLAB and Java programing language implementation. The performance comparison of the MATLAB and the Java 3DMax implementation for a GM06990_HindIII cell line dataset. The Figure shows two different runs of the Java implementation compared against the MATLAB implementation. Models produced by both implementations are comparable with a similar accuracy. Y-axis denotes either the distance Spearman correlation coefficient (dSCC) score in the range [− 1,1]. X-axis denotes the Chromosome number. A higher dSCC value means the better accuracy

To improve the computing performance and the usability of our algorithm, we also implemented the 3DMax algorithm in the Java programming language (available via https://github.com/BDM-Lab/3DMax/releases). The performance comparison of the MATLAB and the Java programming versions for a GM06990_HindIII cell line dataset is shown in Fig. 7. As shown in the Figure, the result produced by two separate Java implementation runs is consistent with those of the MATLAB implementation. We tested 3DMax and all other methods on an Intel Core i5-2400 3.10GHz computer with 8GB RAM.

We compared 3DMax algorithm with the other algorithms mentioned above in terms of computation speed, and the memory cost. To do this, we benchmarked them against the chromosomes of GM06990_HindIII cell data. It takes 3DMax java implementation about 13 s to predict the structure for all the chromosomes of the entire genome when it uses a single conversion factor (α), while it generates a single structure for each chromosome. 3DMax uses about 20 min to generate the representative structures for the entire cell when it estimates the optimal conversion factor (α) in the range [0.1, 2].

Though ChromSDE produced one of the best results, it was memory intensive and slow to generate large structures. ChromSDE could not handle efficiently input data with > 400 bins on our machine with 8 GB RAM. We were only able to use ChromSDE to create structure on our server machine with 65GB RAM. It takes ChromSDE 20-25 h to generate structure for the entire GM06990_HindIII cell data. MOGEN uses over 2 h to generate the models for the cell line. It takes LorDG about 1 h and 7 min to process the whole cell line. MCMC5C with the default parameters uses 1 h and 19 min to generate the models. But to obtain better accuracy by increasing the number of iterations and the number of structures generated, the MCMC5C algorithm could run for > 18 h before it converges.

### Validation using FISH data

**Fig. 8:**
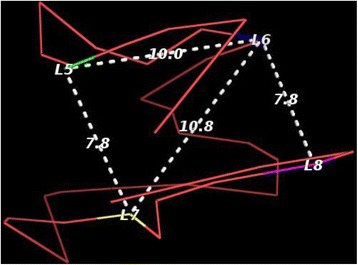
Validation with FISH data. Distances between four fluorescence in situ hybridization (FISH) probes in the model of Chromosome 22 reconstructed by 3DMax. L5, L6, L7 and L8 denote four probes. The distances between the probes are labelled along the virtual line segments connecting the probes

We validated the model of Chromosome 22 reconstructed by 3DMax with an independent FISH data for GM06990_HindIII cell. Four 3D FISH probes for four loci (L5, L6, L7, L8) of the consecutive positions alternate between two chromosome compartments (A and B) [10]. That is, locus L5 and locus L7 are in Compartment A, and locus L6 and locus L8 are in Compartment B. According to the FISH data, L7 is spatially closer to L5 than to L6, though L6 lies between L5 and L7 on the chromosome sequence. Likewise, L6 is spatially closer to L8 than to L7. To check if this holds in the reconstructed 3D model, we measured the distance between these loci on the predicted structure. Figure 8 shows a model constructed by 3DMax with the four probes L5, L6 L7, L8 colored green, blue, yellow, magenta respectively. The distances between these loci: L5 – L6, L5 – L7, L6 – L7, L6-L8 are reported. Indeed, the distance L5 – L7 was shorter than L5 – L6 and the distance L6 - L8 was shorter than L6 – L7. The 3D structure was visualized with Pymol [46].

## Conclusions

We developed a new method (3DMax) based on the maximum likelihood inference to reconstruct the 3D structure of chromosomes from Hi-C data. 3DMax combines a maximum likelihood algorithm and a gradient ascent method to generate optimized structures for chromosomes. The results on synthetic datasets show that the method performs robustly in the presence of noise and structural variability. This method provides a way to automatically determine the best conversion factor (α) for any Hi-C contact data. The results on the real Hi-C datasets reveals that 3DMax can effectively reconstruct chromosomal models from Hi-C contact matrices normalized by different methods. We also show that a major strength of the 3DMax algorithm is that it is faster and has a low memory requirement compared to some other methods.

## Abbreviations

3C: Chromosome Conformation Capture
3DMax: Three dimensional structure modeling using a maximum likelihood approach.
3DMax1: A variant of 3DMax
ICE: Iterative Correction and Eigenvector decomposition
IF: Interaction Frequency
PCC: Pearson Correlation Coefficient
RMSE: Root Mean Square Error
SCC: Spearman’s correlation coefficient
SCN: Sequential Component Normalization
SV: Structural Variability
TAD: Topological Associating Domain
